# O.2.1-6 Effects of test modality and testing site on exercise-induced hypoalgesia in healthy human males: a protocol

**DOI:** 10.1093/eurpub/ckad133.111

**Published:** 2023-09-11

**Authors:** Vladimir Aron, Louise Deldicque, André Mouraux

**Affiliations:** Institute of Neurosciences, UCLouvain, Belgium; vladimir.aron@uclouvain.be; Institute of Neurosciences, UCLouvain, Belgium; vladimir.aron@uclouvain.be; Institute of Neurosciences, UCLouvain, Belgium; vladimir.aron@uclouvain.be

## Abstract

**Background:**

A single session of physical exercise can acutely reduce experimental pain—a phenomenon known as exercise-induced hypoalgesia (EIH). There is inconsistency in the literature as to (1) whether EIH differentially impact the perception of different types of nociceptive stimuli (e.g., pressure versus thermal stimuli) and (2) whether exercise selectively modulates pain at exercising body parts, or also involves non-exercising body parts.

**Purpose:**

We aim to characterize the effects of a single session of aerobic exercise on the sensitivity to stimuli activating skin versus muscle nociceptors, within or outside exercising body parts.

**Methods:**

We will recruit 40 healthy male participants aged 18-30 years. First, we will familiarize them with the sensory assessments and conduct a maximal cycling test. Then, we will assess, in two sessions, the sensitivity to blunt pressure, mechanical pinprick, contact heat and cold, and auditory stimuli before and after a cycling exercise versus a control session. Sensory testing will be performed over the rectus femoris muscle of the dominant leg (local site) and over the flexor muscles of the non-dominant forearm (remote site). EIH will be quantified as the absolute and relative changes in sensitivity to the sensory stimuli before and after exercise.

**Conclusions:**

We hypothesize that EIH will be greater (1) in the exercise than in the control session, (2) at the local site compared with the remote site if local changes in nociceptive sensitivity contribute to EIH, and (3) when blunt pressure stimuli are used if EIH involves specific changes in sensitivity of muscle nociceptors.

